# Proximate Chemical Composition, Fatty Acid Profile, and Lipid Qualitative Indices of Brown Bear Meat

**DOI:** 10.3390/foods10010036

**Published:** 2020-12-24

**Authors:** Nikolina Kelava Ugarković, Miljenko Konjačić, Josip Malnar, Kristijan Tomljanović, Nikica Šprem, Damir Ugarković

**Affiliations:** 1 Department of Animal Science and Technology, Faculty of Agriculture, University of Zagreb, Svetošimunska cesta 25, 10002 Zagreb, Croatia; mkonjacic@agr.hr; 2 Croatian Forests Ltd. Zagreb, Forest Administration Delnice, Forest Office Gerovo, Pilanska 13, 51304 Gerovo, Croatia; Josip.Malnar@hrsume.hr; 3 Institute of Forest Protection and Wildlife Management, Faculty of Forestry, University of Zagreb, Svetošimunska cesta 25, 10002 Zagreb, Croatia; ktomljanovic@sumfak.hr; 4 Department of Fisheries, Apiculture, Wildlife Management and Special Zoology, Faculty of Agriculture, University of Zagreb, Svetošimunska cesta 25, 10002 Zagreb, Croatia; nsprem@agr.hr; 5 Institute of Ecology and Silviculture, Faculty of Forestry, University of Zagreb, Svetošimunska cesta 25, 10002 Zagreb, Croatia; dugarkovic@sumfak.hr

**Keywords:** game meat, lipids, nutritional value, *Ursus arctos*

## Abstract

Although game meat quality has been under the spotlight in numerous studies, the quality of brown bear (*Ursus arctos*) meat is still unknown. The aim of this study was to determine the effects of sex and age on the proximate chemical composition, fatty acid profile, and lipid indices of brown bear meat. Nine (*n* = 9) females and nine (*n* = 9) males were hunted during the Croatian spring hunting period in 2018. Based on age, bears were divided into two groups: <3 years (*n* = 9; five females and four males) and 4–6 years (*n* = 9; four females and five males). For analysis purposes, samples of *M.* *semimembranosus* were collected. Age was shown to have an effect on the traits analyzed, while sex-related differences were not found. Brown bear meat has a high fat content (average 6.12%), especially in older bears (~9%). The contents of protein, dry matter, and ash were similar to those of other game species. Monounsaturated fatty acids made up approximately 50% of all fatty acids, with the most abundant being C18:1n-9. More favorable profiles of essential polyunsaturated fatty acids were found in younger bears. The ratio of polyunsaturated and saturated fatty acids was closer to the recommended ratio than the ratio of n-6 and n-3 polyunsaturated fatty acids, and lipid indices were favorable. Further research is needed to determine seasonal changes in brown bear meat quality.

## 1. Introduction

Game meat differs from the meat of domestic animals in terms of physical and chemical characteristics and nutritional value [1,2]. As game meat is lean, high in protein (20%), low in fat (1–5%), and has a favorable fatty acid profile, it is a good source of valuable nutrients and can have human health promotion benefits [2,3]. 

There have been numerous studies on the meat quality of different game species harvested worldwide, like wild boar (*Sus scrofa*) [4,5,6,7], red deer (*Cervus elaphus*), fallow deer (*Dama dama*) [8,9,10,11,12,13], European mouflon (*Ovis aries musimon*) and axis deer (*Axis axis*) [14,15], impala (*Aepyceros melampus*) and springbok (*Antidorcas marsupialis*) [16,17,18,19], black bear (*Ursus americanus*) [20], and beaver (*Castor fiber*) [21].

However, data regarding the meat quality of certain game species, including brown bear (*Ursus arctos*), are still unavailable. Brown bears are the most widely distributed ursids, inhabiting 45 countries from North America to Europe and northern Asia [22]. In Europe, it inhabits 22 countries, and it is grouped into 10 populations: Scandinavian, Karelian, Baltic, Carpathian, Dinaric-Pindos, Eastern Balkan, Alpine, Central Apennine, Cantabrian, and Pyrenean. According to the IUCN Red List of Threatened Species, the global status of brown bear is of the ˝least concern˝ with a stable population trend and an estimated mature bear population of around 110,000 individuals [23]. The brown bear population in Croatia is part of Dinaric-Pindos, and since 2013, the brown bear has been classified as a highly endangered species, but it is also considered to be a game species managed by legal acts, and each year, an annual hunting quota is determined based on an action plan [24,25,26]. 

In global wild game trading, in addition to brown bear trophies (skin and skull), a reported 17,945 kg of brown bear meat is sold [27]. Bear meat is traditionally consumed by some populations, like the Eastern James Bay Cree people [20], and bear paws are a delicacy in Romania as well as in some other European and Asian countries. Starting from more than 500 hundred BC, bear paws have been considered one of the most precious and sought-after ingredients in Chinese cuisine [28]. In Siberia, traditional dumplings called ˝pelmeni˝ are traditionally made from bear meat [29]. Some USA states (California, Georgia, New Hampshire, Pennsylvania) have prohibited the use of bear meat, but in others, it can be bought online, with the most expensive cuts retailing at 80 euros per pound and the most inexpensive cuts retailing at 15 euros per pound [30]. Bear meat represents a traditional foodstuff for the residents in the Croatian western mountain region Gorski Kotar [31], and it is usually used in stews and as cured and smoked ham.

Meat quality parameters, like fat content and fatty acid profile, are of high importance due to their effects on human health, and game meat is perceived to be a healthier choice than meat from domestic animals [3]. As there are no data regarding brown bear meat quality, the aim of this study was to determine the effects of sex and age on the proximate chemical composition, fatty acid profile, and lipid quality indices of brown bear meat originating from the Croatian Dinaric Mountains.

## 2. Materials and Methods 

All actions in this study were done according to Croatian [24,25,26] and EU legislation [32]. Ethical approval for this study was given by the Bioethical Committee for the Protection and Welfare of Animals of the University of Zagreb Faculty of Agriculture (Croatia) (Class: 114-04/20-03/10; Ref. 251-71-29-02/19-20-1, 05-10-2020).

### 2.1. Study Area, Animals, and Sampling

**Figure 1:**
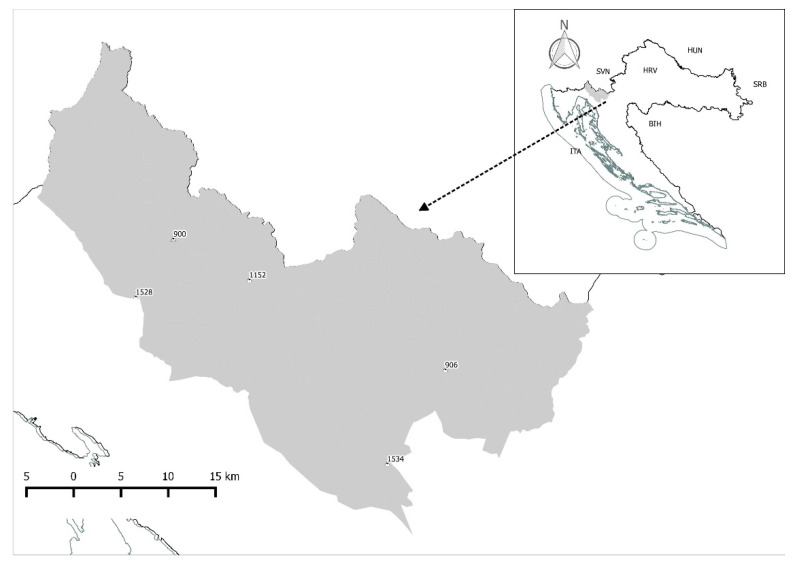
Study area map (Gorski Kotar, Croatia).

The research was conducted in the Gorski Kotar region (Figure 1). Gorski Kotar is a mountainous region in western Croatia with small plateaus and fields lying mainly in the Kupa River Valley. Forests are composed mainly of fir (*Abies alba*) and spruce (*Picea abies*) at elevations between 200 and 1533 m a.s.l. The climate is mountainous with a Mediterranean influence and a mean annual temperature of 7.7 °C and a mean annual rainfall of 2.079 L/m^2^. The Gorski Kotar region is inhabited by four large game species: Wild boar, roe deer (*Capreolus capreolus*), red deer, and Alpine chamois (*Rupicapra r. rupicapra*). Moreover, the region is inhabited by three large carnivore species: Brown bear, wolf (*Canis lupus*), and Eurasian lynx (*Lynx lynx*) [31].

The permanent brown bear habitat in Croatia extents over 9253 km^2^, and sporadic habitats cover 2570 km^2^ [33]. The brown bear population in Croatia consists of about 975 individuals [34], who are managed in accordance with international conventions (Bern Convention), plans, and recommendations (Large Carnivora Initiative for Europe (LCIE) by contract for EC, 2007). The first management plan for brown bear was written in 2005, and the last revision occurred in 2019 [35]. Hunting is allowed only from hunting towers and includes a spring period from February 16 to May 15 and an autumn period from September 16 to December 15 [36]. The planned and allowed hunting quota for the year 2018 was 140 individuals (60% males and 40% females). Commercial use of brown bear meat in Croatia (or export) is allowed after obtaining an EU permit given by the Ministry of Environmental Protection and Energy for each legal and reported bear hunt [36]. 

Meat samples were taken from eighteen brown bears (*n* = 18) hunted during the spring hunting period in 2018. Male and female bears were evenly represented (*n* = 9 per sex group). After skinning, trunks were weighed and the average body weight for males was 101.86 kg (±52.34 Std Dev), and for females, it was 76.83 kg (±26.76 Std Dev). Based on size and weight parameters as well as the degree of tooth wear [37], brown bears were divided into two age groups: Group I—less than three years of age (*n* = 9; four males and five females) and group II—four to six years of age (*n* = 9; five males and four females). Approximately 250 g of the semimembranosus muscle was collected from the trunks, transported in a refrigerated container to the laboratory, vacuum packed, and frozen at −20 °C for chemical and fatty acid analyses. 

Brown bears are classified as true carnivores, however, a feces analysis of brown bears in Croatia showed that 95% of their nutrients are of plant origin [35]. In Croatia, the brown bear diet includes a large variety of indigenous plants, seeds, fruits, insects, and honey [35]. In the early spring, the brown bear diet contains abundant wild garlic (*Allium ursinum* L.), lord and ladies (*Arum maculatum* L.), and grasses (*Graminae* sp.), clovers (*Trifolium* sp.), and sorrels (*Rumex* sp.) from forest meadows. The bears scavenge on dead wild animals and eat the larvae of ants and other insects [35]. Supplement feeding is also done, but only in hunting grounds that have obtained permission to hunt brown bears. Supplement feeding is allowed for a maximum of 120 days annually and can include up to 300 kg of grains (corn, oats, and barley), 300 kg of sugar or fodder beets and other fruits, and 400 kg of animal by-products per adult brown bear. It is not permissible to supplement with by-products of domestic ruminants (material of Category I), however, Category II and III materials including by-products of monogastric domestic animals (pig, poultry), fish, and parts of wild game species can be offered [36]. For the spring period, supplemental feeding of brown bears in Croatia can be done from January 1 to April 30 [35]. Muscle samples used in this study originated from bears that were additionally fed only with corn in amounts determined by Brown Bear Management Plan for the Republic of Croatia [35].

### 2.2. Proximate Chemical Composition Analyses

Before chemical analyses, samples of brown bear, *M. semimembranosus*, were thawed, and all excessive and visible adipose tissue was removed. Samples were then minced, mixed, and homogenized. To determine the dry matter content, 5 g of each muscle sample was put in an aluminum container with 2–3 g of quartz sand. In the following step, 2 mL of ethanol (96%) was added and sand was scrubbed into the samples. Drying was done at 103 °C for 4 h. Next, samples were cooled in a desiccator and then weighed [38].

To determine the fat content of the brown bear meat samples, 5 g of muscle was put into a 400 mL beaker. Pumice stone and 50 mL of the HCl solution (4 M) were added to the beaker. The content of the beaker was mixed and placed on a hot plate to boil for one hour. After this time, the content was poured over filter paper, rinsed with water, and stored overnight. The next day, filter paper was placed in the extraction thimble, and extraction was performed using hexane for four hours. When evaporation of the hexene was completed, drying was performed in a preheated oven at 98 ºC, and samples were weighed [39].

The protein content was determined from 1 g of each muscle sample weighed in a glass tube used for Kjeldahl analysis. To the tube, 13 mL of concentrated sulfuric acid was added, and samples were digested for one hour at 420 °C. Once completely cooled, according to the Kjeldahl method, the protein content was determined using a protein analyzer FOSS Kjeltec 8400 (Hilleroed, Denmark) [40].

In a crucible, 5 g of muscle sample was weighed and placed in an oven heated at 100 ºC for pre-drying. Samples were then placed in a furnace at 550 ºC for four hours and turned to ash. When cooled, weighing was done, and the ash content was determined [41].

### 2.3. Determination of the Fatty Acid Composition 

In brown bear muscle samples, the fatty acid methyl ester (FAME) content was determined by gas chromatography [42]. Sample methylation was performed using a saturated sodium–chloride solution. FAMEs were quantified on a Shimadzu GC2010 gas chromatograph (Shimadzu Corp., Kyoto, Japan). The chromatograph was equipped with a CP-Sill 88 silica capillary column (100 m length, 0.25 mm wall coated open tubular-WCOT, 0.2 µm, Varian, Santa Clara, CA, USA). Analyses were performed under a temperature program ranging from 130 to 202 °C. The temperature of the injector and detector was maintained at 270 °C. The relative FAME peak retention times of each sample were compared, and using fatty methyl ester standards from Supelco (Supelco 37 Component Fame Mix 47885-U, Sigma Aldrich, St.Louis, MO, USA), individual fatty acids were identified. Fatty acids were expressed as percentages of each individual fatty acid peak area relative to the total of all fatty acids present in the sample. 

### 2.4. Calculation of Lipid Quality Indices

The atherogenicity index (*AI*) and thrombogenicity index (*TI*) were calculated as lipid quality indices in accordance with Ulbricht and Southgate [43]. Using sums of hypocholesterolemic (*h*) and hypercholesterolemic (*H*) fatty acids, the h/H ratio was calculated in accordance with Santos-Silva et al. [44]. The peroxidability index (*PI*) was calculated in accordance with Du et al. [45].

### 2.5. Statistical Analysis

The Shapiro–Wilk test was used to measure the distribution and variance homogeneity of samples using SAS Software (Cary, NC, USA) [46]. A one-way ANOVA was used to analyze data with a normal distribution, while the Kruskal–Wallis test was used to test nonparametric data. The significance level was set at *p* < 0.05. Interactions between sex and age groups were analyzed, however, the model parameters (*F* value, *p* value) of the statistical model were the same as for individual variables. Therefore, we decided not to present interactions in the results. Body weight was included in the model as a covariate. The results are presented as the mean ± SE (standard error).

## 3. Results

### 3.1. Proximate Chemical Composition

Sex had no effect (*p* > 0.05) on the brown bear meat proximate chemical composition, whereas differences were found between age groups (Table 1). Meat samples originating from younger age (group I) brown bears had less (*p* < 0.05) fat and a lower (*p* < 0.05) moisture content. 

### 3.2. Fatty Acid Composition

The individual saturated fatty acid (SFA) content in the brown bear meat was not affected (*p* > 0.05) by either sex or age group (Table 2). The dominant SFA in the analyzed brown bear meat samples was C16:0 with a very similar content in males and females, as well as in younger (group I) and older (group II) bears. The second most prevalent SFA was C18:0 with a similar content between sex and age groups. The same was found for C14:0, which was the third most prevalent SFA. Other SFAs in brown bear meat were found in contents lower than 1% (Table 2). 

The content of the dominant monounsaturated fatty acid (MUFA), C18:1n-9, was higher (*p* < 0.005) in the meat of older bears than that in younger bears. The content of C16:1, the second-most abundant MUFA, did not differ (*p* > 0.05) between sex or age groups (Table 2). 

The most abundant PUFA in analyzed brown bear meat samples was C18:2n-6, with similar contents between sex and age groups. Several PUFAs showed significant differences between age groups. Namely, the contents of C20:3n-6, C20:5n-3, C22:5n-3, and C22:6n-3 were higher (*p* < 0.05) in younger than in older bears. Moreover, younger bears had higher (*p* < 0.005) C20:4n-6 contents than older bears. (Table 2). 

Sex showed no effect on the total sums of fatty acids (Table 3). In meat of older bears, a higher (*p* < 0.05) content of MUFA was found, but there were lower (*p* < 0.05) PUFA and PUFAn-6 contents. The polyunsaturated/saturated fatty acids ratio (PUFA/SFA) was higher (*p* < 0.05) in meat from younger than older bears (Table 3).

In this study, effect of sex on lipid indices was not found (*p* > 0.05), and AI, PI and h/H had similar values between sex groups (Table 4). PI was lower (*p* < 0.005) in meat from older compared with younger bears. Brown bear meat from both age groups had similar AI and h/H values. 

## 4. Discussions

This is the first study to present the brown bear meat quality. Although the brown bear is protected and managed by special acts in many countries, it is also a game species that is hunted for trophies, and meat is available for human consumption. Therefore, data presenting the proximate chemical composition and fatty acid content could be beneficial for consumers and a broader audience. 

In the present study, the fat content in brown bear meat was higher than that reported for other omnivores [4,5,6,7] and herbivore wild game species [8,9,10,11,12,13,14,15,16,17,18,19]. The fat content in meat is highly variable and influenced by many factors, especially diet [1]. The higher meat fat content in brown bears compared with other species could be species-related and attributed to different diets and specific fat metabolism characteristics due to hibernation, i.e., wintering. The protein, moisture, and ash contents were similar to those of roe deer [9], red and fallow deer [10,47,48,49], impala [16], and springbok [19]. 

As in the present study, no effect of sex on proximate chemical composition was reported in previous studies that analyzed wild boar meat [1,50,51,52] and meat from fallow and red deer [13,48,49] and springbok [19]. Differences in the proximate chemical composition between brown bear age groups, especially fat content, correspond to those found for mouflon [14], springbok [19], fallow deer [47,53], wild boar [51], and red deer [54]. 

It can be concluded that brown bear meat has more fat than other wild game species, but similar moisture, protein, and ash contents. A higher fat content can be expected in meat originating from older brown bears, while sex-related differences were not found. 

The content of dominant SFAs (C16:0 and C18:0) found in this study was similar to that found in previous studies on black bear [20] and wild boar meat [4,5,7]. However, Rolinec et al. [6] reported higher amounts of dominant SFAs in wild boar meat. Lower C16:0 and C18:0 contents were reported in the subcutaneous adipose tissue (SAT) of female brown bears, while in the SAT of male brown bears, a similar C16:0 content but a higher C18:0 content was found [55]. The content of odd-fatty acids (C15:0 and C17:0) found in brown bear meat was similar to or lower than that reported for wild boar [4,5], wild and farmed red deer [48], fallow deer [49], and roe deer and wild boar [50]. 

For decades, C16:0, C14:0, and C12:0 have been associated with cardiovascular diseases, primarily causing increased LDL and cholesterol levels. However, C18:0 has not been associated with an increased incidence of such health-related problems. The general recommendation was to limit the dietary SFA content to 10%. This was mainly due to an increased share of consumed processed food, which contains a high proportion of SFAs. The C16:0 content found in brown bear meat should not be considered as a potential nutritional disadvantage. Moreover, dietary SFAs originating from natural sources and unprocessed meat are suggested to be health-neutral [56]. C16:0 in brown bear meat during the spring period mainly originates from fresh green vegetation, like *Allium* species, lord and ladies [57,58], which are preferred by brown bears. Supplementary feeding with corn could have also contributed to C16:0 content [59] in brown bear meat.

As in studies on wild boar [4,5,7], black bear [20], fallow deer [47,53] and red deer [54], C18:1n-9 was found to be the most abundant MUFA in brown bear meat. The C18:1n-9 content found in brown bear meat is similar to that reported for wild boar [4,5,6,7] and roe deer [9] but lower than that reported for springbok [19], roe deer and wild boar [50], fallow deer [49,53], and red deer [48,54]. Black bear meat is also characterized by a similar C18:1n-9 content [20]. Vranković et al. [55] reported a lower C18:1n-9 content in male brown bear SAT, while in female SAT samples, almost the same C18:1n-9 content was found as in meat. In brown bear meat, a high content of C18:1n-9 as a dominant MUFA is beneficial from a nutritional point of view. Generally, meat from all species is a good source of MUFAs known to lower the incidence of arteriosclerosis and reduce the level of cholesterol. Green spring vegetation (*Allium* species, lords and ladies) can be considered to be the main source of C18:1n-9 in meat in the bears studied here. Supplementary feeding (corn) could have also contributed to C18:1n-9 content in meat. 

The content of C18:2n-6 found in brown bear meat was similar to that reported for roe deer [9], fallow and red deer [48,53]. A higher C18:2n-6 content than that found in brown bear meat was reported for wild boar [7], springbok [19], fallow deer [47], roe deer and wild boar [49,50], red deer [54], and black bears [20]. Besides species-related, dietary differences can be considered as the main reason for different C18:2n-6 content in meat of game species. Brown bear meat had less favorable C18:2n-6 content, probably due to less dietary available C18:2n-6, and further research is needed to determine possible seasonal changes.

Contrary to the present study, a minor effect of age on the content of essential fatty acids, like C20:3n-3, C20:4n-6, C20:5n-3, and C22:6n-3, was reported in wild boar [4,5], fallow deer [47], and red deer [54] meat. Same as in other game species, in brown bear meat, C20:4n-6 was the second most abundant PUFA. This content was lower than that reported for wild boar hunted in Tuscany and Lithuania [4,7], springbok [19], red deer [48], and fallow deer [49], but higher than that found in wild boar hunted in Slovakia [5], fallow deer [47], and red deer [54]. Besides species-related differences, these differences can mainly be attributed to differences in diet. The profile of PUFA in brown bear meat is affected by age, and a greater content of essential PUFAs was found in younger brown bears. Sex-related differences regarding the content of essential PUFAs were not found. 

A similar content of SFA, a higher MUFA content, and a lower PUFA content were reported for black bear meat [20]. Vranković et al. [55] reported similar values of SFA, MUFA, and PUFA in brown bear SAT, with no sex-related differences. No changes in fatty acid sums related to age were reported for wild boar [6], while in fallow deer [47] and red deer [54] a decrease in fatty acid sums with age was reported. Compared to other game species, brown bear meat has a high MUFA content (~50%), and this is most similar to the content reported for wild boar [6,7]. Brown bears have a lower PUFA content than that reported for fallow deer [47,49] and red deer [48,54]. However, very similar PUFA contents to brown bear meat were reported for roe deer [9] and fallow deer [53]. Rolinec et al. [6] reported a much lower PUFA content in wild boar meat than that found in this study. It seems that regardless of species, dietary sources of fatty acids can greatly affect the game meat fatty acid composition.

Fatty acid ratios (PUFA/SFA and n-6/n-3) and lipid indices (AI, TI, PI and the h/H ratio) can be used to evaluate the nutritional value of dietary fat sources. The recommendation is that PUFA/SFA ratio should be >0.40 [60] and the n-6/n-3 ratio should be < 4.0 [60]. Regarding PUFA/SFA, brown bear meat is within the recommended values, especially the meat of younger bears. However, brown bear meat has a less preferable n-6-/n-3 ratio, especially the meat from male and older bears. Contrary to this study, no effect of age on PUFA/SFA was reported in wild boar [4]. No effect of age on the n-6/n-3 content was reported for fallow deer [47,49] and wild boar [50], while, as in this study, age-related differences were reported for wild boar [4], mouflon [14], and red deer [54]. Compared to other species, the PUFA/SFA content in brown bear meat was similar to that reported in wild boar meat [4,7] but higher than that reported for roe deer [9] and fallow deer [47,53]. 

The lipid indices, AI, and TI for brown bear meat were < 1, as recommended, and the h/H ratio was at a favorable value of > 2.5 [61]. Similar h/H ratio values to those found in this study were reported for wild boar meat [4,7]. Compared to the PI values found in this study, higher values were reported for wild boar [4,7], beaver meat [21], free-living and farmed red deer [48]. This indicates a lower potential for the peroxidation of brown bear meat, which has comparable PI values to those of lard or poultry lipids [62]. 

## 5. Conclusions

The fatty acid profile, ratios, and lipid indices of brown bear meat are characterized by a high content of C18:1n-9 and, consequently, a high MUFA content, a better essential PUFA profile in younger individuals, and a good PUFA/SFA ratio. The high meat fat content and less favorable n-6/n-3 ratio can be identified as nutritional disadvantages. In general, brown bear meat can be compared to wild boar meat, which is one of the most available game meats. However, further research, including research on brown bear meat samples harvested during the autumn period (priory to hibernation), is needed to highlight seasonal effect on analyzed traits. 

## Figures and Tables

**Table 1:** Proximate chemical composition of the brown bear, *M. semimembranosus*, as influenced by sex and age groups (mean ± SE).

Parameter (%)	Sex		*P*-Value	Age		*P*-Value
	Male (*n* = 9)	Female (*n* = 9)		Group I (*n* = 9)	Group II (*n* = 9)	
Moisture	71.40 ± 1.19	72.15 ± 1.28	0.676	74.00 ± 0.90	69.81 ± 0.83	0.006
Protein	20.00 ± 0.25	20.18 ± 0.27	0.653	20.37 ± 0.25	19.84 ± 0.23	0.550
Total fat	7.26 ± 1.31	6.37 ± 1.41	0.626	4.32 ± 0.98	9.01 ± 0.91	0.045
Ash	1.17 ± 0.06	1.18 ± 0.07	0.903	1.20 ± 0.07	1.16 ± 0.06	0.659

**Table 2:** Fatty acid composition of the brown bear, *M. semimembranosus*, as influenced by sex and age groups (mean ± SE).

Fatty Acids (%)	Sex		*P*-Value	Age		*P*-Value
	Male (*n* = 9)	Female (*n* = 9)		Group I (*n* = 9)	Group II (*n* = 9)	
C12:0	0.02 ± 0.00	0.02 ± 0.00	0.634	0.02 ± 0.00	0.02 ± 0.00	0.332
C14:0	1.22 ± 0.10	1.18 ± 0.09	0.822	1.23 ± 0.11	1.26 ± 0.10	0.819
C15:0	0.26 ± 0.02	0.31 ± 0.04	0.287	0.29 ± 0.04	0.30 ± 0.04	0.812
C16:0	23.10 ± 0.72	23.34 ± 0.56	0.797	23.11 ± 0.75	23.13 ± 0.71	0.980
C17:0	0.48 ± 0.03	0.56 ± 0.05	0.199	0.52 ± 0.06	0.54 ± 0.05	0.809
C18:0	8.11 ± 0.52	8.35 ± 0.44	0.735	7.96 ± 0.61	8.38 ± 0.50	0.625
C20:0	0.26 ± 0.02	0.26 ± 0.03	0.829	0.29 ± 0.02	0.24 ± 0.02	0.139
C22:0	0.06 ± 0.01	0.06 ± 0.01	0.889	0.08 ± 0.01	0.06 ± 0.01	0.347
C14:1	0.19 ± 0.03	0.20 ± 0.02	0.736	0.22 ± 0.03	0.19 ± 0.02	0.529
C16:1	4.19 ± 0.51	3.73 ± 0.23	0.433	4.36 ± 0.49	3.86 ± 0.46	0.478
C18:1n-9	42.79 ± 1.13	41.19 ± 1.45	0.397	40.38 ± 1.23	44.22 ± 1.14	0.004
C18:1n-7	2.92 ± 0.16	2.80 ± 0.20	0.656	2.81 ± 0.19	2.72 ± 0.18	0.724
C20:1	1.13 ± 0.23	1.13 ± 0.13	0.989	1.07 ± 0.23	1.23 ± 0.22	0.640
C22:1	0.13 ± 0.03	0.13 ± 0.02	0.913	0.14 ± 0.02	0.13 ± 0.02	0.613
C18:2n-6	11.43 ± 1.12	11.80 ± 0.87	0.797	12.11 ± 1.24	10.80 ± 1.14	0.455
C18:3n-6	0.06 ± 0.01	0.06 ± 0.01	0.938	0.06 ± 0.01	0.06 ± 0.02	0.919
C18:3n-3	0.66 ± 0.08	1.20 ± 0.52	0.317	1.35 ± 0.47	0.72 ± 0.44	0.351
C20:2n-6	0.21 ± 0.02	0.16 ± 0.02	0.078	0.17 ± 0.02	0.20 ± 0.02	0.420
C20:3n-6	0.29 ± 0.04	0.28 ± 0.03	0.882	0.32 ± 0.03	0.21 ± 0.03	0.015
C20:3n-3	0.03 ± 0.01	0.04 ± 0.01	0.640	0.04 ± 0.01	0.04 ± 0.01	0.928
C20:4n-6	1.85 ± 0.28	2.20 ± 0.47	0.536	2.39 ± 0.22	1.24 ± 0.20	0.003
C20:5n-3	0.06 ± 0.01	0.11 ± 0.03	0.107	0.12 ± 0.03	0.05 ± 0.02	0.050
C22:5n-3	0.46 ± 0.07	0.74 ± 0.17	0.131	0.79 ± 0.13	0.34 ± 0.12	0.027
C22:6n-3	0.10 ± 0.01	0.12 ± 0.02	0.465	0.15 ± 0.02	0.08 ± 0.01	0.025

**Table 3:** Fatty acid sums in the brown bear, *M. semimembranosus*, as influenced by sex and age groups (mean ± SE).

Sums of Fatty Acids (%)	Sex		*P*-Value	Age		*P*-Value
	Male (*n* = 9)	Female (*n* = 9)		Group I (*n* = 9)	Group II (*n* = 9)	
SFA	33.51 ± 0.90	34.09 ± 0.72	0.619	33.50 ± 1.02	33.94 ± 0.95	0.758
MUFA	51.35 ± 0.84	49.19 ± 1.39	0.204	48.99 ± 1.09	52.34 ± 1.01	0.046
PUFA	15.14 ± 1.30	16.72 ± 1.23	0.389	17.50 ± 1.24	13.72 ± 1.14	0.050
PUFA n-6	13.84 ± 1.21	14.50 ± 1.07	0.688	15.05 ± 1.18	12.51 ± 1.03	0.043
PUFA n-3	1.30 ± 0.10	2.21 ± 0.60	0.158	2.45 ± 0.53	1.21 ± 0.50	0.116
PUFA/SFA	0.46 ± 0.05	0.49 ± 0.04	0.612	0.52 ± 0.05	0.41 ± 0.04	0.031
n-6/n-3	10.75 ± 0.71	8.69 ± 1.23	0.170	8.59 ± 1.27	10.39 ± 1.18	0.320

SFA = sum of saturated fatty acids, MUFA = sum of monounsaturated fatty acid, PUFA = sum of polyunsaturated fatty acids, n-3 PUFA= sum of n-3 PUFA, n-6 PUFA = sum of n-6 PUFA, n-6/n-3 = n-6 and n-3 PUFA ratio.

**Table 4:** Lipid quality indices of the brown bear, *M. semimembranosus*, as influenced by sex and age groups (mean ± SE).

Lipid Indices	Sex		*P*-Value	Age		*P*-Value
	Male (*n* = 9)	Female (*n* = 9)		Group I (*n* = 9)	Group II (*n* = 9)	
AI	0.42 ± 0.02	0.43 ± 0.01	0.849	0.42 ± 0.02	0.43 ± 0.02	0.825
TI	0.89 ± 0.03	0.86 ± 0.03	0.497	0.82 ± 0.04	0.91 ± 0.04	0.111
h/H	2.38 ± 0.13	2.35 ± 0.08	0.811	2.36 ± 0.13	2.38 ± 0.12	0.931
PI	26.27 ± 2.17	31.23 ± 3.36	0.236	33.26 ± 1.98	22.24 ± 1.83	0.002

AI = atherogenic index, TI = thrombogenicity, h/H = hypocholesterolemic/hypercholesterolemic ratio, PI = peroxidizability index.
